# Tensile behavior of Cu-coated Pd_40_Cu_30_Ni_10_P_20_ metallic glassy wire

**DOI:** 10.1038/s41598-018-23956-5

**Published:** 2018-04-04

**Authors:** I. Hussain, Y. Y. Jiang, Y. D. Jia, G. Wang, Q. J. Zhai, K. C. Chan, J. Yi

**Affiliations:** 1 Laboratory for Microstructures, Institute of Materials, Shanghai University, Shanghai, 200444 China; 2 Department of Chemistry, Karakoram International University, Gilgit-Baltistan, 15100 Pakistan; 3 Department of Industrial and System Engineering, The Hong Kong Polytechnic University, Hong Kong, China

## Abstract

Catastrophic brittle fracture of monolithic metallic glass (MG) hinders engineering application of MGs. Although many techniques has been tried to enhance tensile ductility of metallic glasses, the enhancement is quite limited. Here, we show the effect of electrodeposited Cu coating on tensile plasticity enhancement of Pd_40_Cu_30_Ni_10_P_20_ MG wires, with different volume fractions of copper coatings (*R*), from 0% to 97%. With increasing *R*, tensile elongation is enhanced to 7.1%. The plasticity enhancement is due to confinement of the Cu coatings, which lead to multiple and secondary shear bands, according to SEM investigations. In addition, the SEM images also show that the patterns on the fracture surface of the Cu-coated MG wires vary with volume fraction of the Cu coatings. The size of shear offset decreases with increasing *R*. The viscous fingerings on the fracture surface of monolithic MG wire changes into dimples on the fracture surface of Cu coated MG wires with *R* of 90% and 97%. The electrodeposition technique used in this work provides a useful way to enhance plasticity of monolithic MGs under tensile loading at room temperature.

## Introduction

Plasticity of bulk and micro-sized MG is mediated by shear bands with highly localized strain^1,2^. However, in tension, the tensile stress enhances softening and instability, a dominant shear band slips without obstacle, and bulk and micro-sized MGs fracture elastically^3^. Various approaches have been introduced to retard the dominant shear band to improve tensile ductility of bulk and micro-sized MGs, such as designing MG matrix composites^4,5^, sharp-and-deep notches^6^, laminating MG with ductile crystalline metals^7,8^, surface mechanical treatment^9–11^, laser surface texturing treatment^12^, and MG-based chiral nanolattice^13^. In addition, size reduction approach changes room temperature plastic deformation mechanism of MG from shear banding into homogeneous plastic flow^14–18^. However, the transition occurs at submicron length scale^17,19^.

At the same time, other techniques have been introduced to improve compressive plasticity of bulk MGs. What attracted our attention was metal coating technique. By using this technique, compressive plasticity of Fe-based metallic glass was improved from 0.5% to 5.0%^20^. Furthermore, a theoretical study has shown that a thin metal coating slows down shear band dynamics and retards its attainment to a critical unstable state^21^. However, metal coating effects on tensile plasticity of MGs have not yet been investigated.

Here, Pd_40_Cu_30_Ni_10_P_20_ MG wires are chosen for investigation, because such wires can have much higher ratio of lateral surface area to cross section area that provide sufficient adhesive force to prevent sliding between the MG core and the metal coating, as described in the literature^21^. Cu is chosen to be electrodeposited onto the Pd_40_Cu_30_Ni_10_P_20_ MG wires for investigation, because the Cu electrodeposition technique is flexible and facile. It is shown that tensile ductility of the Cu coated MG wires increases significantly with increasing Cu coating content. At the same time, fracture morphology changes from viscous fingering to dimpling.

## Results

### Coating quality

**Figure 1:**
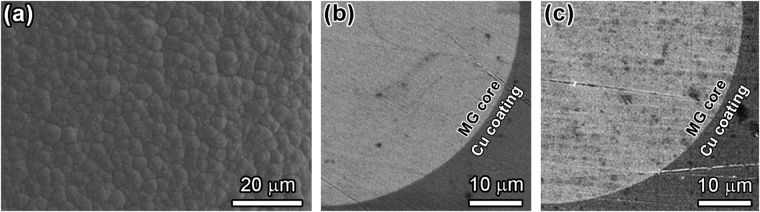
Surface and cross-sectional morphology of Cu-coated Pd_40_Cu_30_Ni_10_P_20_ MG wires: (**a**) optical image of the cross section of Cu-coated Pd_40_Cu_30_Ni_10_P_20_ MG wire; (**b**) SEM image of the surface of homogeneous Cu coating without detected defects.

High quality coating is expected for high mechanical performance of metal coated materials^8^. Therefore, before tensile tests, the coating quality was investigated using scanning electron microscope (SEM). The SEM images in Fig. 1 show surface (Fig. 1(a)) and the cross-sectional (Fig. 1(b)) of morphology a Cu-coated Pd_40_Cu_30_Ni_10_P_20_ MG wire. Figure 1(a) shows homogeneous Cu coating surface without any detected defects. At the same time, the cross-sectional image of the coated wire in Fig. 1(b) shows that the Cu layer is continuous without pore; and no defects, such as voids and/or cracks, can be detected at or near the interface between the coating and the MG core. All these images confirm that high quality Cu coating was well adhered to the MG wire. The high quality electrodeposition was kept from sample to sample by using constant electrodeposition parameters, such as electrolyte composition, working temperature, current density, pH value, the surface state of the MG wire, electroplating time span and so forth by following previous work^22^. The high quality can also prevent the MG core from sliding during tension. In order to confirm this, the two ends of the sample shown in Fig. 5(b) was polished by using SiC paper along the dashed lines before tensile testing. After the tensile testing, the polished cross-sections were investigated in SEM. The SEM image in Fig. 1(c) shows no sliding can be detected along the interface between the Cu coating and the MG core.

### Tensile behavior of as-cast and Cu-coated MG wires

**Figure 2:**
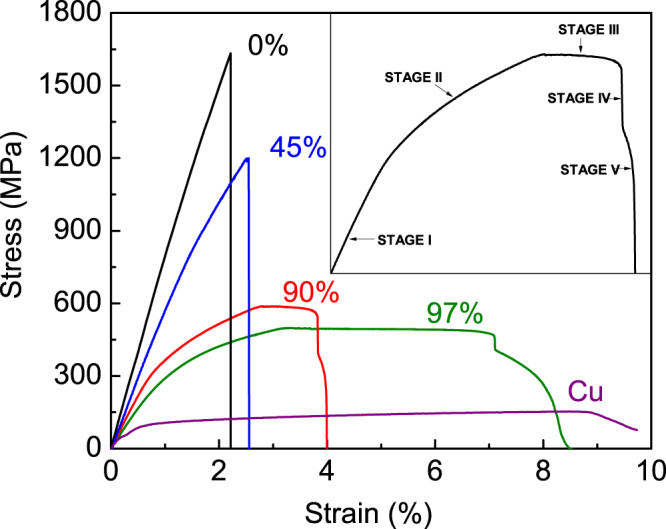
Engineering tensile stress-strain curves of Cu-coated Pd_40_Cu_30_Ni_10_P_20_ MG wires with coating volume fraction *R* of 0%, 45%, 70%, 90%, and 97%, and electrodeposited pure Cu. The straight dash lines are eyesight guide of the tensile stress-strain curves of the as-cast Pd_40_Cu_30_Ni_10_P_20_ MG wire and the wire with *R* of 45% for showing the nonlinearity of the curve. The inset indicates five stages of tensile deformation process of Cu-coated Pd_40_Cu_30_Ni_10_P_20_ MG wires. In stage I, both the coating and the wire core deform linearly. In stage II, the coating deforms plastically while the wire core elastically. In stage III, both the coating and the wire core deform plastically. In stage IV, the wire core fractures. In stage V, the coating necks and then fractures.

Tensile behavior of as-cast and Cu-coated Pd_40_Cu_30_Ni_10_P_20_ MG wires with specific *R* values of 45%, 90%, and 97% were characterized as shown in Fig. 2 by using tensile testing sample as shown in Fig. 5(b).

The stress-strain curve of as-cast wires shows that it fractures catastrophically without any plasticity as bulk metallic glasses^3^ and other MG wires^11,23–25^. The deformation of the as-cast wire shows nonlinearity before fracture while bulk metallic glasses do not^3^. The nonlinearity has been investigated both experimentally and theoretically^23^ and is outside the scope of this work.

The stress-strain curve of the wire with *R* value of 45% also shows nonlinearity. Here, the nonlinearity is mostly from plasticity of Cu coating. Nevertheless, Young’s modulus *E* of pure Cu measured from tensile stress-strain curve is much smaller than that measured by using acoustic technique because plasticity of pure copper initiates at a very low strain value^26^. That’s why the slope of the linear region of stress-strain curves of wires with *R* value of 45% is obviously smaller than that of as-cast Pd_40_Cu_30_Ni_10_P_20_ MG wires. According to tensile stress-strain curve in literature^26^, the elastic deformation region and theoretical yield strength cannot be defined. Therefore, *E* of the Cu-coated Pd_40_Cu_30_Ni_10_P_20_ MG wires cannot be calculated as volume-weighted *E* of the coating and the MG core as that of composites reinforced by aligned continuous fiber^27^. Furthermore, 0.2% offset yield strength can be defined as 941 MPa as listed in Table 1. The wire with *R* value of 45% fracture catastrophically at a strain of 2.55% and a strength of 1199 MPa. The fracture strain is increased by 0.34% while the strength is reduced. The stress-strain curve also shows that the Cu coating and the MG core fracture simultaneously. However, this is different from compressive fracture of Cu-coated bulk metallic glass, during which the Cu coating completely peers off the metallic glass core^21^. In a word, the tensile deformation and fracture behavior of the Cu-coated Pd_40_Cu_30_Ni_10_P_20_ MG wires with a *R* value of 45% is very similar to fiber reinforced composites with high fiber volume fraction as reported in literature^27^.

**Table 1:** Yield strength ${\sigma }_{y}$, tensile strength ${\sigma }_{T}$, strength at the fracture of MG wire ${\sigma }_{f,w}$, strain at the fracture of MG wire ${\varepsilon }_{f,w}$, fracture strength ${\sigma }_{f}$, and fracture strain ${\varepsilon }_{f}$ of Cu-coated Pd_40_Cu_30_Ni_10_P_20_ MG wires with different *R* values.

*R*	${\sigma }_{y}$ (MPa)	${\sigma }_{T}$ (MPa)	${\sigma }_{f,w}$ (MPa)	${\varepsilon }_{f,w}$ (%)	${\sigma }_{f}$ (MPa)	${\varepsilon }_{f}$ (%)
0%	1633 ± 24	1633 ± 24	1633 ± 24	2.21 ± 0.322	1633 ± 24	2.21 ± 0.32
45%	941 ± 17	941 ± 17	1198 ± 21	2.49 ± 0.34	1198 ± 21	2.49 ± 0.34
70%	484 ± 16	900 ± 17	873 ± 14	2.55 ± 0.26	873 ± 15	2.55 ± 0.31
90%	371 ± 10	588 ± 13	547 ± 13	3.82 ± 0.21	260 ± 11	3.98 ± 0.20
97%	311 ± 9	498 ± 10	464 ± 9	7.09 ± 0.24	0	8.50 ± 0.18

When *R* increases to a value of 70%, the tensile behavior of the wire changes as shown in Fig. 2. With increased *R* value, the yield strength ${\sigma }_{y}$ decreases to 484 MPa as listed in Table 1. At the same time, the tensile strength ${\sigma }_{T}$ and fracture strength ${\sigma }_{f}$, which are 900 MPa and 873 MPa (Table 1), respectively, do not equal to each other anymore. Therefore, strain localization happens before fracture because the tensile strength is higher than the fracture strength^28^. Furthermore, the MG core and the Cu coating of the wire still fracture concurrently as that of the wire with lower *R* value described above.

When the *R* value increases to 90%, the tensile stress-strain curve in Fig. 2 shows that deformation and fracture behavior of the wire varies a lot and is very similar to tensile stress-strain curve of aligned continuous metal fiber reinforced composites with lower fiber volume fraction^27^. In order to clearly describe the uniaxial stress-strain response, the curve is divided into five stages as shown in the inset of Fig. 2. In stage I, both the coating and the MG core deforms linearly. When it comes to stage II, the MG core continuously deforms elastically, because strain range of stage II is within the elastic limit of monolithic MG. At the same time, the Cu coating deforms plastically, because the relationship between stress and strain is no longer linear. In stage II, the stress decreases slightly with increasing strain, and the strain range goes outside the elastic limit of monolithic Pd_40_Cu_30_Ni_10_P_20_ MG wires. As reported in the literature^29^, monolithic MG undergoes softening during plastic deformation. Therefore, the plateau in the stress-strain curve indicates plastic deformation of Pd_40_Cu_30_Ni_10_P_20_ MG core. This will be further discussed in the next section. Near the end of stage III, the stress drops faster with increasing strain. Then, the stress drops abruptly and the MG core fractures in stage IV. However, the coating and the MG core do not fracture concurrently. Therefore, Cu coating deforms plastically and then fractures in stage V. As shown in Fig. 2, the deformation behavior of the wire with *R* value of 97% is very similar to that of the wire with *R* value of 90%. Their difference is that the stress and strain values are different as listed in Table 1. The 0.2% offset yield strength is reduced from 371 MPa to 311 MPa. Surprisingly, the fracture strain of the Pd_40_Cu_30_Ni_10_P_20_ MG core is increased from 3.82% to 7.09% during uniaxial tensile. This indicates that metallic glassy wires can provide not only high strength but also significant ductility in fiber reinforced composites.

### Deformation and fracture morphology

#### Transition from catastrophic shear fracture to ductile fracture

**Figure 3:**
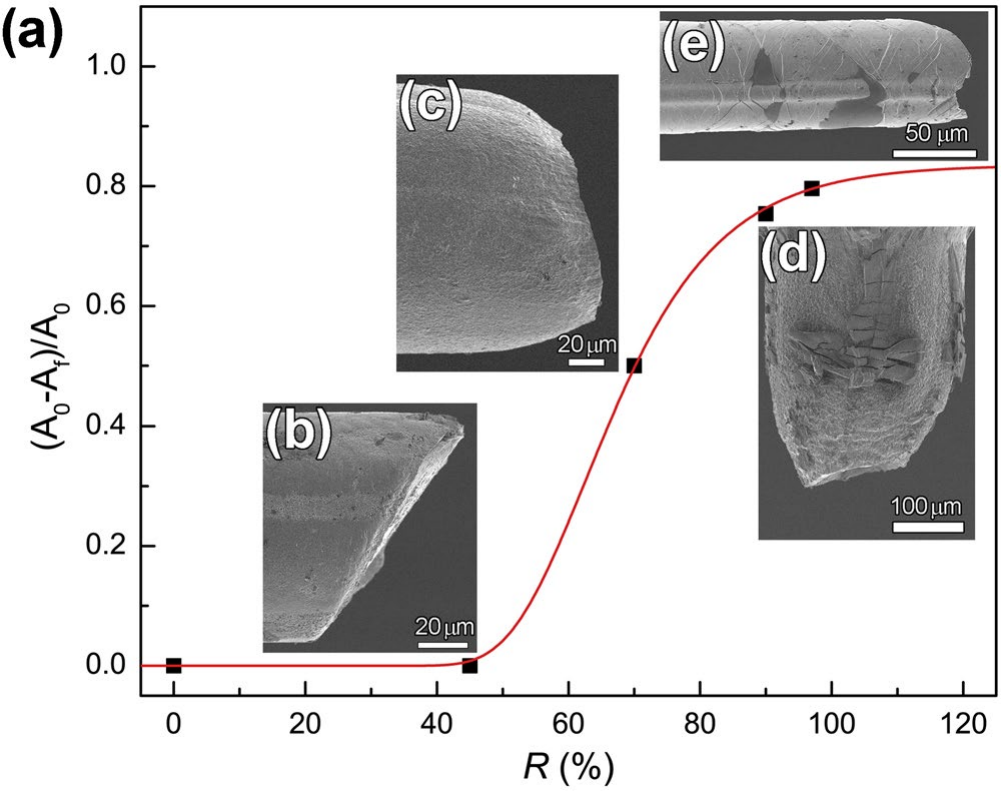
Catastrophic shear fracture to necking transition of Cu-coated Pd_40_Cu_30_Ni_10_P_20_ MG wires. (**a**) Variation of the reduction of area $({A}_{0}-{A}_{f})/{A}_{0}$ with volume fraction of Cu coating *R*. (**b**,**c** and **d**) are fracture morphology of Cu-coated Pd_40_Cu_30_Ni_10_P_20_ MG wires with *R* of 45%, 70% and 90%, respectively. (**e**) Morphology of the MG core of the Cu-coated Pd_40_Cu_30_Ni_10_P_20_ MG wires with *R* of 97%.

Not only as-cast MG wire but also the wires with low *R* value, such as the wire with *R* value of 45%, fracture catastrophically as shown in Fig. 3a and b. When the *R* value increases to 70%, the reduction of area $({A}_{0}-{A}_{f})/{A}_{0}$ (where ${A}_{0}$ is the cross-sectional area and ${A}_{f}$ is the cross-sectional area of the fracture) increases to 50%, even though the ductility improvement is almost zero when it is compared with the MG wire with *R* value of 45%. Therefore, the transition from catastrophic shear fracture to ductile necking of the MG wires happens somewhere between *R* values of 45% and 70%. However, Fig. 3c shows that the final fracture still happens in shear. When the *R* value further increases to 90%, $({A}_{0}-{A}_{f})/{A}_{0}$ increases to 75%, and the final fracture shown in Fig. 3d changes totally into ductile manner. The Morphology of the MG wire core electropolished from the wire with *R* value of 97% is shown in Fig. 3e. The image indicate that the ductility of the MG wire core is mediated by dense shear banding like compressive ductility of bulk metallic glasses^30^. However, no indication of necking can be found on the MG wire core. This transition from catastrophic shear fracture to ductile failure is the transition from catastrophic shear fracture to ductile necking of MG wires with reduction in wire diameter as reported in our previous work^17^. Nevertheless, the 7% tensile elongation mediated by dense shear banding was not expected because zero tensile ductility of microscale MGs had been well founded in literatures^1,31^. The dense shear bands in Fig. 3e may be caused by retarding effect on shear band dynamics as in compressed bulk MG with Cu coating^20–22^.

#### Transition from viscous fingering to dimpling

**Figure 4:**
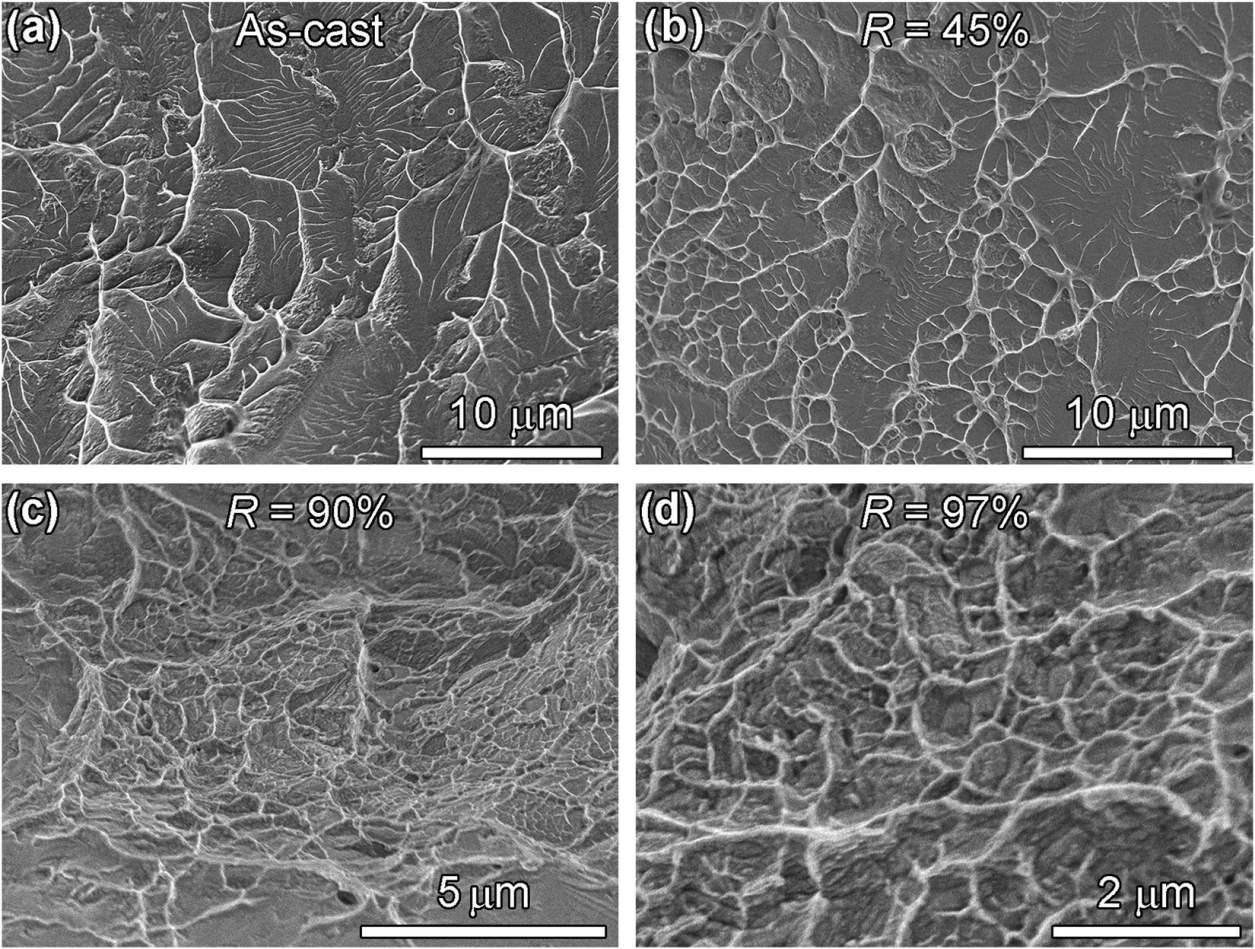
Fracture patterns on the fracture surface of Pd_40_Cu_30_Ni_10_P_20_ MG core: (**a**) viscous fingering on the fracture surface of as-cast Pd_40_Cu_30_Ni_10_P_20_ MG wire; (**b**) viscous fingerings and dimples on the fracture surface of the MG wire core of the wire with a *R* value of 45%; (**c**,**d**) dimples on the fracture surface of the MG wire cores in the wires with *R* values of 90% and 97%.

In addition to the transition described above, transition from viscous fingering to dimpling was observed on fracture surfaces of the wires with different *R* values as shown in Fig. 4. As on fracture surfaces of various as-cast MG wires^25,32–34^, viscous fingering was found on the fracture surface of as-cast Pd_40_Cu_30_Ni_10_P_20_ MG wires as shown in Fig. 4a. The viscous fingering is an indication of Mode II fracture^35,36^ and a continuous crack propagation manner^37^. On the fracture surface of the MG wire core of the Cu-coated wire with an *R* value of 45%, both viscous fingering and dimples were found as shown in Fig. 4b, that is, the viscous fingering is changed into dimples at an *R* value around 45%. Even though the wire fractures in catastrophic shear as shown in Fig. 3b, the MG wire core is confined by the Cu coating. Therefore, the final fracture of the wire is not in pure shear mode, and there may be a contribution of normal stress which causes the formation of dimples. With increasing *R* value, the fracture surfaces become rougher as shown in Fig. 4c and d, and the stress states on the fracture surfaces of the MG wire cores become more complex. The rough surface would be caused by the intersecting shear bands showing in Fig. 3e, because the wires can no longer fracture along a single shear band. At the same time, the average dimple size of the wires with *R* values of 90% and 97% becomes smaller. Because of the rough fracture surfaces and complex stress states, the facture patterns shown in Fig. 4c and d cannot be analyzed theoretically by using physical models.

## Discussion

In order to investigate the effect of Cu coating on the tensile behavior of Cu-coated Pd_40_Cu_30_Ni_10_P_20_ MG wires, the tensile behavior of the coating was tested as shown in Fig. 2. The elastic limit is much lower than that of the as-cast MG wire, and the stress increases slowly with strain. Furthermore, Poisson’s ratio *ν* of metals undergo plastic deformation is 0.5^38^. Therefore, we just simply take the Poisson’s ratio *ν* of the Cu coating as 0.5 to simplify theoretical analysis. In addition, *ν* of Pd_40_Cu_30_Ni_10_P_20_ MG is 0.399^39^. The inner diameter of a tube or the diameter of a rod $d={d}_{0}(1-\nu \varepsilon )$, where ${d}_{0}$ is the original diameter, *ε* is the tensile strain. Hence, the inner diameter of a Cu tube is smaller than the diameter of a Pd_40_Cu_30_Ni_10_P_20_ MG rod with the same diameter ${d}_{0}$. Therefore, there is pressure which is perpendicular to the loading direction and increases with increasing *R*. The pressure causes yielding strain of the MG core to increase with increasing *R* as shown in Fig. 3, because dependence of yielding strength of MG on pressure is negligible^40,41^. However, a literature^42^ has reported that plasticity of BMGs can be improved by pressure. This also applies to our experimental results that ductility increases with increasing pressure which increases with increasing *R*, as shown in Fig. 2. Because of the pressure, we believe that the dislocations in Cu coating sink at the Cu-MG interface, and a high volume fraction of crystalline phase means a high number of dislocation therefore a higher propensity for glassy phase to yield.

The tensile behavior of Cu-coated Pd_40_Cu_30_Ni_10_P_20_ MG wire with different volume fraction of Cu coating *R* has been investigated. A maximum tensile elongation of 7.1% of the MG core has been achieved. SEM investigation has revealed that the tensile ductility is induced by multiple shear banding. Fracture of the wires transit from catastrophic shear fracture to ductile fracture at an *R* value around 45%. At the same time, the pattern on the fracture surface of the MG core transits from viscous fingering to dimple. The pressure imposed on the surface of the MG core, which is induced by the Poisson’s ratio difference between the MG core and the Cu coating, is the reason of the ductility and the transitions. The pressure generated during deformation may be an effective way to tailor strength and ductility of materials.

## Methods

### Preparation of Cu-coated MG wires

The details of the preparation process of Pd_40_Cu_30_Ni_10_P_20_ MG wire can be found in our previous work^24^. The diameters of the prepared Pd_40_Cu_30_Ni_10_P_20_ MG wires range from 50 ∼ 100 μm.

**Figure 5:**
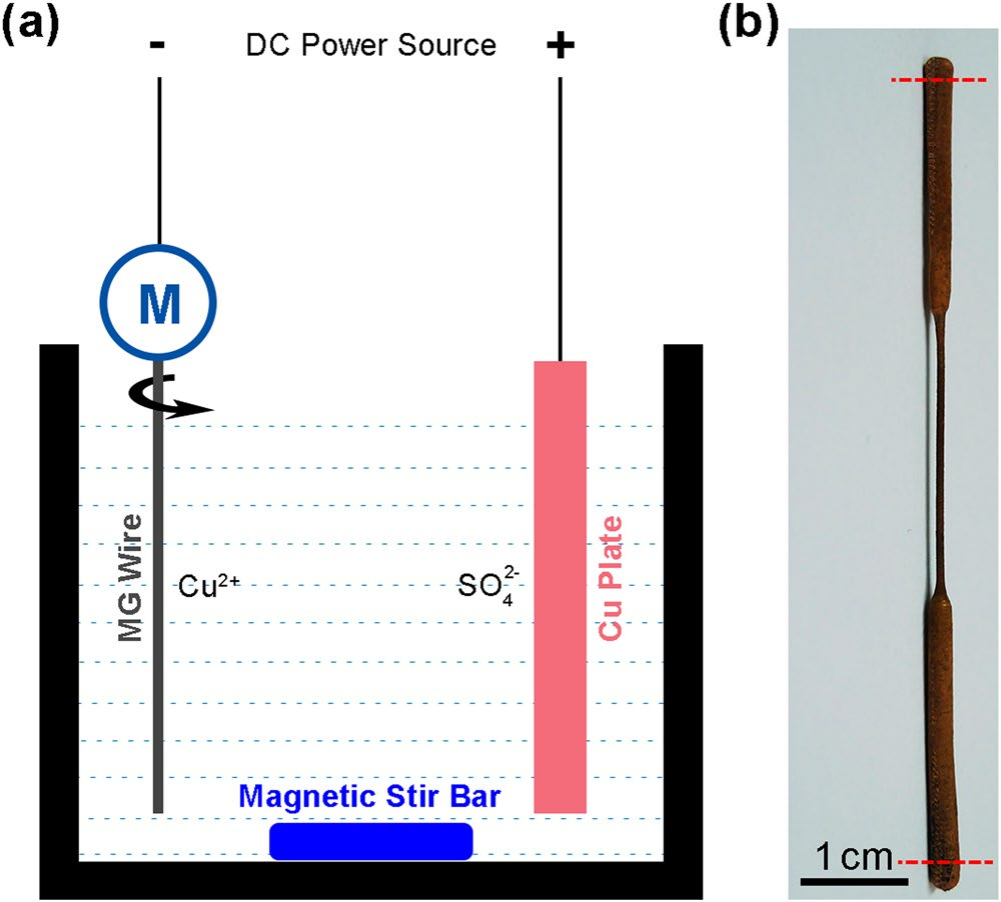
Electrodepositing Cu onto Pd_40_Cu_30_Ni_10_P_20_ MG wire for tensile testing. (**a**) Schematic illustration of electrodeposition setup. The difference between our setup and conventional one for Cu electrodeposition is that a motor is connected to the cathode to rotate the MG wire to make the coating thickness homogeneous. (**b**) Cu coated Pd_40_Cu_30_Ni_10_P_20_ MG wire for tensile testing. The fillet between the grip section and the reduced section of the prepared tensile testing sample forms during electrodeposition because of the meniscus electrolyte around the MG wire at the position where the wire meets the surface of the electrolyte.

The electrolyte for Cu electrodeposition was prepared from copper sulfate pentahydrate (AR), sulfuric acid with a purity of 98 wt.%, and distilled water with 64 g/L Cu ion and 73.5 g/L sulfuric acid. The setup for Cu electrodeposition onto Pd_40_Cu_30_Ni_10_P_20_ MG wires is schematically shown in Fig. 5(a). A DC power supply was used to provide constant current. The anode was a copper-phosphorous (phosphorous content about 0.1 wt.%) plate with dimensions of 2 mm × 10 mm × 85 mm, and the cathode was a Pd_40_Cu_30_Ni_10_P_20_ MG wire. In order to ensure constant coating thickness, the cathode wire was clamped to the shaft of the motor as shown. The cathode was spinning at a speed of about 100 rpm during electrodeposition. The distance between the anode and cathode was kept at 30 mm. A magnetic stirrer with a spinning speed of about 120 rpm was used to stir the electrolyte. The current density *i* used in this work was 1 mA/mm^2^. The constant current provided by the power supply is *I* = *iπd*_0_*l*, where *d*_0_ is the diameter of the as-cast Pd_40_Cu_30_Ni_10_P_20_ MG wires, *l* is the length of the wire immersed in the electrolyte as shown in Fig. 5(a). In this work, the value of *l* was kept at a constant value of 70 mm.

In our experiments, the volume fraction of the Cu coating, given by $R=1-{d}_{0}^{2}/{d}_{1}^{2}$ (where *d*_*1*_ is the diameter of the Cu coated wires), needs to be controlled to specific values. The *R* was controlled by setting the electrodeposition time *t* on the power supply. The volume of electrodeposited metal is proportional to *I·t*^43^, therefore, we used a commercial Ni wire with a diameter of 0.175 mm to measure the volume of Cu electrodeposited per second. The current *I*_0_ was calculated to be 0.038 A by using the equation above. After one-hour electrodeposition, the thickness of the wire became 0.321 mm. Then, the electrodeposition rate *C*_0_ was calculated to be 1.4 × 10^−3^ mm^3^/s. Therefore, the time needed to achieve a specific *R* value is *t = Rπd*_0_^2^*lI*_0_*/4I(1* − *R*).

After electrodeposition of Cu with expected *R*, two end parts with lengths of 25 mm were electrodeposited with extra Cu to produce tensile testing sample shown in Fig. 5(b). When the surfaces of the wire meet the surface of the electrolyte, a meniscus of electrolyte forms around the wire. This is how the fillet forms between the grip section and the reduced section of the tensile sample, as shown in Fig. 5(b). Because of the fillet, the tensile testing sample fractured near the middle of the reduced section.

In order to test tensile properties of the Cu coating, Cu layers with thickness of 0.6 mm were electroplated onto pure Al plates with dimensions of 2 mm × 10 mm × 70 mm with the same electroplating parameters described above. After electroplating, the Al plates were removed by using a NaOH solution, and the Cu layers were machined into dog-bone tensile testing specimen, with gauge dimensions of 0.6 mm × 3 mm × 15 mm.

### Electropolishing of Cu-coating

In order to investigate the deformation morphology of the MG core of the Cu-coated MG wires, the Cu-coating was electropolished before SEM observation. The setup in Fig. 5a was used for electropolishing. However, the Cu-coated sample was set as the anode and the Cu plate was set as the cathode. The electrolyte used was the same as the electrolyte for electrodeposition. The motor did not spin during the electrodeposition. The power supply was working in the constant voltage mode, with a voltage of 0.5 V to electropolish the Cu Coatings. At this low voltage, the MG core cannot be electropolished.

### Mechanical, structure and morphology tests

The fully amorphous nature of the as-cast Pd_40_Cu_30_Ni_10_P_20_ wires was confirmed on a micro-diffractometer with Cu-K_α_ radiation. Tension tests were conducted at room temperature at a strain rate of 1 × 10^−3^ s^−1^ on an Instron 3345 machine. The Fracture surface morphology was investigated using an SEM.

### Data availability

The data in this work are available from the corresponding authors on reasonable request.
